# Abiotic degradation of chlorinated ethanes and ethenes in water

**DOI:** 10.1007/s11356-012-0764-9

**Published:** 2012-02-01

**Authors:** Marek Tobiszewski, Jacek Namieśnik

**Affiliations:** Department of Analytical Chemistry, Chemical Faculty, Gdańsk University of Technology (GUT), ul. G. Narutowicza 11/12, 80-233 Gdańsk, Poland

**Keywords:** Chlorinated ethanes and ethenes, Reductive dechlorination, Abiotic degradation, DNAPL

## Abstract

**Introduction:**

Chlorinated ethanes and ethenes are among the most frequently detected organic pollutants of water. Their physicochemical properties are such that they can contaminate aquifers for decades. In favourable conditions, they can undergo degradation. In anaerobic conditions, chlorinated solvents can undergo reductive dechlorination.

**Degradation pathways:**

Abiotic dechlorination is usually slower than microbial but abiotic dechlorination is usually complete. In favourable conditions, abiotic reactions bring significant contribution to natural attenuation processes. Abiotic agents that may enhance the reductive dechlorination of chlorinated ethanes and ethenes are zero-valent metals, sulphide minerals or green rusts.

**Oxidation:**

At some sites, permanganate and Fenton’s reagent can be used as remediation tool for oxidation of chlorinated ethanes and ethenes.

**Summary:**

Nanoscale iron or bimetallic particles, due to high efficiency in degradation of chlorinated ethanes and ethenes, have gained much interest. They allow for rapid degradation of chlorinated ethanes and ethenes in water phase, but they also give benefit of treating dense non-aqueous phase liquid.

## Introduction

Chlorinated ethanes and ethenes are produced in great amounts in nature (van Pée and Unversucht 2003), most of which are produced by biota in the marine environment (Gribble 2003). One of the main sources is marine algae, but also abiotic sources such as volcanoes and biomass burning produce considerable emissions. However, some chlorinated derivatives of ethane and ethene are not produced naturally in the environment, e.g. 1,2-dichloroethane (1,2-DCA). Even tetrachloroethene (PCE) and trichloroethene (TCE), which for years were considered to be compounds of anthropogenic origin only, can be produced by seaweed (Ballschmiter 2003). Also, vinyl chloride (VC) may be produced naturally in reaction of humic acids, iron (III) and chlorides (Keppler et al. 2002).

Chlorinated ethanes and ethenes are used as degreasing and cleaning agents, paint removers and industrial solvents, also in the production of pesticides, electronic components and polymers. VC can be leached from PVC piping (Walter et al. 2011). Their improper use and disposal leads to unintentional releases to the environment. Chlorinated ethanes and ethenes are therefore among the most frequently detected pollutants in groundwater in the USA (Moran et al. 2007).

There are regulations stipulating maximum allowable concentrations of chlorinated ethanes and ethenes in surface and drinking waters. In the USA, they are regulated by the Environmental Protection Agency, in Europe by EU Directives; there are also such regulations in WHO guidelines on drinking water quality. The EU Water Framework Directive identifies 1,2-DCA as a “priority” substance, and PCE and TCE as “dangerous” (Lepom et al. 2009).

Table 1 summarises the basic physicochemical parameters of chlorinated ethanes and ethenes. Because of their low boiling points, they are volatile. Formation of dense non-aqueous phase liquid (DNAPL) is possible owing to their poor solubility in water and the fact that the density of most chlorinated ethanes and ethenes is much greater than that of water. Highly chlorinated ethanes and ethenes are less soluble and denser.

**Table 1:** Physicochemical parameters of chlorinated ethenes and ethanes, and the acronyms used in the text

Name	Acronym	Boiling point (°C)	Solubility in water at 25°C (mg L^−1^)	Density at 20°C (g cm^−3^)	EPA maximum contaminant levels (μg L^−1^)
Chloroethane	CA	12.3	5,700	0.89	–
1,1-Dichloroethane	1,1-DCA	57.3	5,060	1.18	–
1,2-Dichloroethane	1,2-DCA	85.3	8,600	1.25	5
*cis*-Dichloroethene	c-DCE	60.1	3,500	1.28	70
1,1-Dichloroethene	1,1-DCE	31.6	2,250	1.21	7
*trans*-Dichloroethene	t-DCE	48.7	6,260	1.26	100
Tetrachloroethene	PCE	121.3	200	1.62	5
Trichloroethene	TCE	88	1,000	1.46	5
1,1,1,2-Tetrachloroethane	1,1,1,2-TeCA	130.2	1,100	1.54	–
1,1,2,2-Tetrachloroethane	1,1,2,2-TeCA	145.2	2,850	1.59	–
1,1,1-Trichloroethane	1,1,1-TCA	74	1,490	1.34	200
1,1,2-Trichloroethane	1,1,2-TCA	113.8	4,390	1.44	5
Vinyl chloride	VC	−13.9	2,700	0.91	2

As degradation products may pose a greater environmental hazard than the parent compounds, knowledge of the degradation patterns is important for reliable risk assessments at contaminated sites. Knowledge is also required of natural attenuation, which is the sum of processes leading to a decrease in pollutant concentrations at a contaminated site. Understanding the degradation pathways of chlorinated ethanes and ethenes in water is crucial in the context of implementing or not implementing remediation, and in the former case, which process to choose. Since remediation techniques are expensive and invasive, natural attenuation is often considered a cost effective but long-lasting technique. The remediation of large numbers of contaminated sites is not feasible, so understanding natural attenuation mechanisms enables one to choose which sites need remediation and which can be left to self-purify naturally (Stiber et al. 2004).

The main issues of chlorinated ethanes and ethenes biodegradation have been reviewed: anaerobic microbial bioremediation (Aulenta et al. 2006), the microbial degradation of chloroethenes (Bradley 2000), the biodegradation of a great variety of chlorinated aliphatic compounds in natural and engineered systems (Field and Sierra-Alvarez 2004), the aerobic biodegradation of *cis*-dichloroethene (c-DCE) and VC (Mattes et al. 2010), and the biodegradation of TCE (Pant and Pant 2010).

The aim of the paper is to review possible abiotic natural and remediation degradation pathways of chlorinated ethanes and ethenes in water. It focuses on factors enhancing or interfering with natural attenuation, and also on agents that are applied in enhanced (or stimulated) natural attenuation or in situ remediation. Abiotic aerobic and anaerobic processes occurring in surface and groundwater systems are reviewed.

## Degradation pathways

The behaviour of chlorinated ethanes and ethenes in natural waters is variable and depends strongly on environmental conditions. For example, at some TCE-contaminated sites a rapid decrease in concentration from high to negligible levels is observed, whereas at others the level of contamination remains rather constant (Chapman et al. 2007). The natural attenuation of chlorinated ethanes and ethenes present in surface and groundwaters may involve physical, chemical or biological processes (Nobre and Nobre 2004). Physical changes include volatilization and sorption on suspended matter and bottom sediments. Therefore, sorption on natural organic matter is applied as a remediation technology (Wei and Seo 2010). However, chlorinated ethanes and ethenes sorbed onto bottom sediments can be slowly released back to water (Riley et al. 2010).

**Fig. 1:**
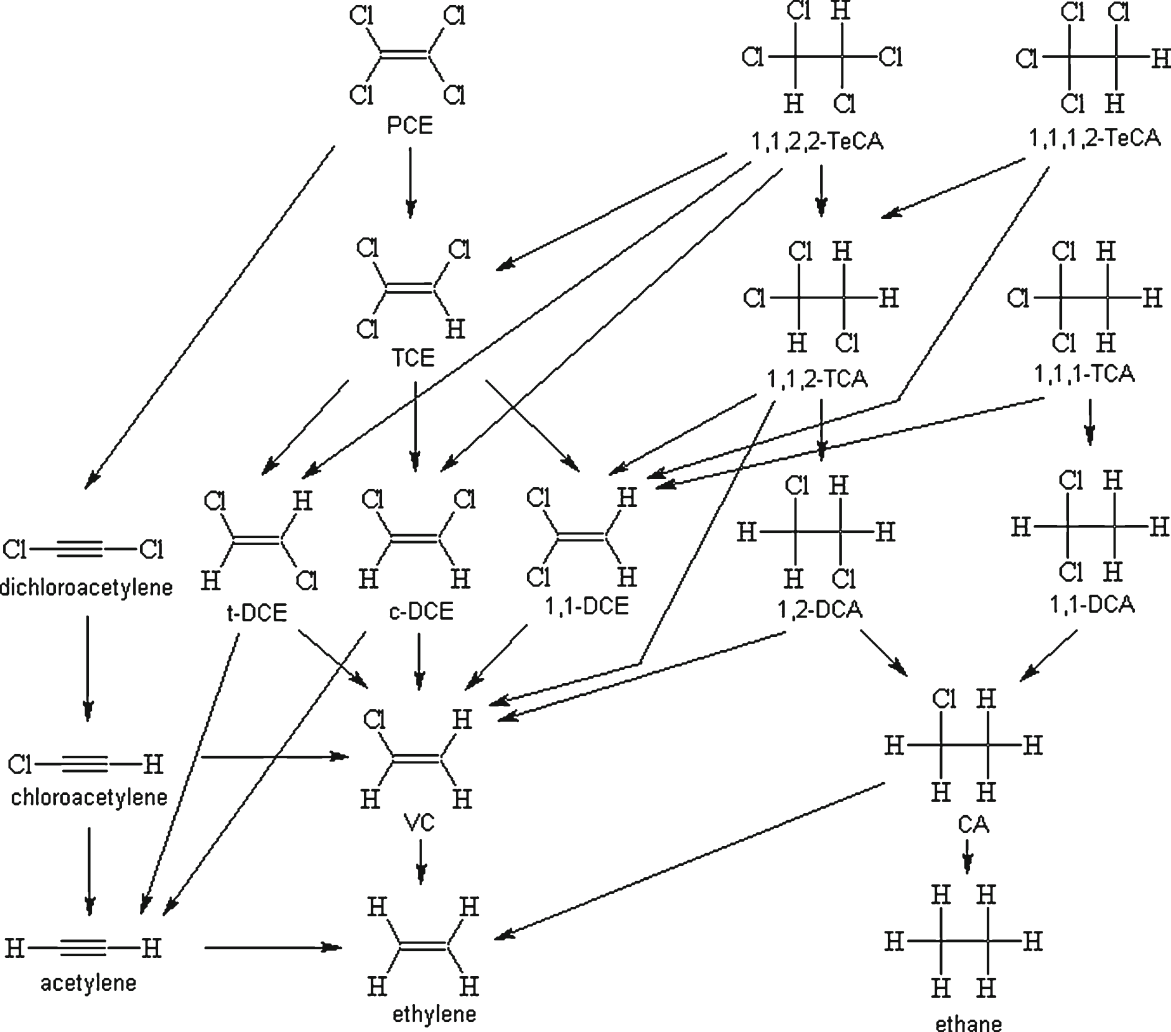
Transformation pathways of chlorinated ethenes and ethanes in anaerobic conditions. Based on O’Loughlin and Burris (2004), Hara et al. (2005), Aulenta et al. (2006), Mattes et al. (2010)

The main dechlorination pathways of chlorinated ethanes and ethenes are presented in Fig. 1. The geochemical, microbiological conditions and co-contaminant patterns in natural surface and ground waters are very complex, so water may exhibit different potential to degrade chlorinated ethenes and ethanes. In environmental conditions, both abiotic and biotic degradation take place. Under anaerobic conditions, microbial degradation is more rapid; however, abiotic transformation may be crucial if the concentration of reactive minerals is high and/or the activity of dechlorinating bacteria is low. Abiotic transformation is significant as the transformation of chlorinated ethanes and ethenes is often nearly complete (Dong et al. 2009). Temperature plays important role in abiotic natural degradation. 1,1,1-trichloroethane (1,1,1-TCA) degradation half-life decreases two times with 5°C increase in temperature in the temperature range 15–25°C (Wing 1999).

The main reaction mechanisms of the dechlorination of chlorinated ethanes and ethenes are hydrolysis, dehydrochlorination, hydrogenolysis and dichloroelimination. Hydrolysis in natural waters is an extremely slow process, though slightly faster in basic conditions.

1 $$ {\text{Hydrolysis}}:{\text{RCl}} + {{\text{H}}_{{2}}}{\text{O}} \to {\text{ROH}} + {\text{HCl}} $$

Chlorinated alkanes may undergo dehydrogenation, in which HCl is eliminated from the solvent molecule. The reaction results in the formation of less saturated and less halogenated compounds. It is not a redox reaction.

2 $$ {\text{Dehydrogenation}}:{\text{RHCCl}} - {\text{CR}}{{\text{H}}_{{2}}} \to {\text{RHC}} = {\text{CHR}} + {\text{HCl}} $$

Hydrogenolysis (reductive dechlorination) is a reductive process, in which a halogen is substituted by a hydrogen atom, with the simultaneous addition of two electrons to the molecule (Mohn and Tiedje 1992). It is the principal degradation pathway for highly chlorinated ethene derivatives (Nobre and Nobre 2004).

3 $$ {\text{Hydrogenolysis}}:{\text{RCl}} + {{\text{H}}^{ + }} + {2}{{\text{e}}^{ - }} \to {\text{RH}} + {\text{C}}{{\text{l}}^{ - }} $$

Dichloroelimination (vicinal reduction, β elimination or α elimination) is a process involving a two-electron transfer to the molecule and the elimination of two chlorine atoms. The reaction products are less saturated aliphatic hydrocarbons and two chloride ions. β elimination occurs when chlorine atoms are removed from two different carbons, whereas α elimination is the elimination of chlorine atoms from one carbon atom. Dichloroelimination occurs mainly under methanogenic conditions but may also take place under partially aerobic conditions (Chen et al. 1996).

4 $$ {\text{Dichloroelimination:RCCl}} - {\text{CClR}} + {2}{{\text{e}}^{ - }} \to {\text{RC}} = {\text{CR}} + {\text{2C}}{{\text{l}}^{ - }} $$

The degradation pathway and rate depend strongly on redox conditions in the groundwater or surface water. Reductive dechlorination takes place in anaerobic conditions.

## Reductive degradation

### Zero-valent iron

Zero-valent iron is used in engineered degradation systems and may contribute to natural attenuation (Cundy et al. 2008). Fe^0^ is capable of degrading chlorinated ethanes and ethenes through reductive dechlorination. The main advantages of Fe^0^ are its low cost and its ability to dechlorinate chlorinated organics over a wide concentration range to non-chlorinated products, like ethane, ethylene and acetylene. The main drawback is the formation of iron oxides and other corrosion by-products on the surface of iron, which may prevent further dechlorination reactions (Ma and Wu 2008), but in acidic conditions corrosion by-products are water soluble (Beverskog and Puigdomenech 1996). The dechlorination reactions are heterogeneous, so the rates depend on the specific surface area of the iron present in water. Since the dechlorination of highly chlorinated ethenes to ethene is a step-wise reaction, intermediates (c-DCE, t-DCE, VC) can be present at low concentrations. Chlorinated ethenes are adsorbed on the iron surface, and sorption is the rate controlling step (Janda et al. 2004). The saturation of active sites on an iron surface results in the increase of reaction half-lives and deviation from a first-order reaction (Farrel et al. 2000) The weak character of sorption of chlorinated ethanes and ethenes can result in a short residence time on the metal surface and desorption of incompletely dechlorinated compounds (Zhang et al. 2008). Therefore, minor amounts of less chlorinated ethylene may be formed during reductive dechlorination on metal surfaces (Orth and Gillham 1996). However, even VC can be reductively dechlorinated via hydrogenolysis in the presence of a high iron concentration (Deng et al. 1999). There are large differences in dechlorination reaction rates, which depend on the kind of iron, particle size and specific surface area, as well as metal storage and pre-treatment conditions (Cheng and Wu 2000).

The main degradation pathway of chlorinated ethenes in the presence of Fe^0^ is β elimination, while reductive dechlorination by means of hydrogenolysis is usually slower. Hara et al. (2005) report that TCE, c-DCE, t-DCE conversions occur 40, 10 and 100 times faster respectively via β elimination than via hydrogenolysis. t-DCE reacts faster than c-DCE in the presence of Fe^0^ because of the more favourable β elimination reaction for *trans* than *cis* isomers (Roberts et al. 1996). 1,1-Dichloroethane (1,1-DCA) undergoes slow degradation in contact with metals (Fennelly and Roberts 1998). 1,2-DCA does not react with zero-valent iron, but reactive iron barriers may support removal of this compound by *Dehalobacter* species. The corrosion of iron causes the release of hydroxyl ion, thus increase of pH which contributes to the enrichment of 1,2-DCA reducing bacteria (Zemb et al. 2010).

1,1,1-TCA is reduced by different types of iron, without chlorinated by-products for some types of iron or 1,1-DCA and chloroethane (CA) as by-products for another type of iron (Lookman et al. 2004).

The fewer chlorine atoms a compound contains, the slower the iron-mediated dechlorination reaction rate (Scherer et al. 1998). This is only partially confirmed by Hara et al. (2005), who investigated TCE dechlorination in the presence of iron powder with a large specific surface area. Dechlorination reactions are enhanced in the presence of metal amendments. Nickel is characterised by a high enhancement of PCE dechlorination; cobalt and copper show similar behaviour but the enhancement is much less (Doong and Lai 2006).

Humic acids, which are ubiquitous in water systems, show inhibitory potential towards dechlorination mediated by metal-modified iron. Doong and Lai (2005; 2006) explain this phenomenon by the competition of humic substances with chlorinated ethanes and ethenes to reach iron active sites. The conclusions of other study are that complexes of humic acids with nickel and copper are active electron transfer mediators in reductive dechlorination in the presence of a reductant (O’Loughlin et al. 1999). TCE was rapidly and almost completely reduced to ethylene: in the presence of microorganisms the iron-mediated TCE dechlorination products are shifted. Compared to iron systems more VC and cDCE were produced, proving that microbial and abiotic degradation may occur simultaneously (Lampron et al. 2001). Under anaerobic conditions, iron reacts with water to form hydrogen gas. Hydrogen is electron donor, needed to support growth of dechlorinating bacteria, including *Dehalococcoides*, the only organisms that dechlorinate solvents completely (Aulenta et al. 2006). 1,1,2,2-Tetrachloroethane (1,1,2,2-TeCA) in the presence of iron is transformed to c-DCE and t-DCE (with two to three times more *cis* isomer) via dichloroelimination and to TCE via dehydrochlorination (Arnold et al. 2002). Nitrates (100 mg L^−1^) inhibited TCE reduction, which was explained by the oxidising effect of nitrates and the enhanced formation of iron oxides on the iron surface (Ritter et al. 2003).

### Zero-valent zinc

Zero-valent zinc has a greater reducing power than Fe^0^: the standard reduction potential of Zn^0^ is −0.763, that of Fe^0^ is −0.44 (Cheng and Wu 2000). Ma and Wu (2008) reported that the half-life of the degradation reaction of PCE in the presence of Zn^0^ is three times shorter than in the presence of Fe^0^. What is interesting is that in the presence of a microbial community, dechlorination occurred faster in the systems containing Fe^0^ than those with Zn^0^. It was explained by stronger interactions of Zn than Fe with microbial community and higher Zn toxicity. The reaction rates of PCE with Zn^0^ are much faster than for daughter products containing fewer chlorine atoms, probably due to favourable dichloroelimination of PCE by Zn^0^ over other compounds (Roberts et al. 1996). The presence of hydroxyapatite (Ca_5_(PO_4_)_3_OH) at amount of 0.01 g of 0.1 g for 1 g of Zn, resulted in increase of PCE reduction by Zn by 150% and 960%, respectively (Song et al. 2008). The authors identified TCE and t-DCE, (not c-DCE as in the case of iron, although the production of the *cis* isomer is thermodynamically favoured over the *trans* isomer) as the degradation products of PCE. 1,1-DCE and VC were not detected during 192 h experiments either. During PCE transformation, dichloroacetylene and chloroacetylene (only chloroacetylene in the case of TCE) are produced via dichloroelimination (Arnold and Roberts 1998). Dichloroacetylene undergoes further rapid reaction to t-DCE and chloroacetylene, and chloroacetylene in turn to acetylene or VC. c-DCE and t-DCE are degraded mostly via dichloroelimination, in contrast to PCE and TCE, which react mostly via hydrogenolysis. Reductive elimination of PCE has great environmental importance, as the production of TCE is avoided, which reacts to produce DCEs and further to VC. Arnold and Roberts (1998) conclude that although only 15% of PCE is transformed via dichloroelimination, this reaction pathway results in the avoidance of toxic VC formation. The main transformation pathways of chlorinated ethanes are presented in Table 2.

**Table 2:** Observed products and reaction pathways for chlorinated ethane reaction by Zn^0^

Parent	Product	Reaction type
HCA	PCE	Reductive β elimination
PCA	TCE	Reductive β elimination
	PCE	Dehydrohalogenation
1,1,1,2-TeCA	1,1-DCE	Reductive β elimination
1,1,2,2-TeCA	*cis*-DCE	Reductive β elimination
	*trans*-DCE	Reductive β elimination
1,1,2-TCA	VC	Reductive β elimination
1,2-DCA	Ethylene	Reductive β elimination
1,1,1-TCA	Ethane	Reductive α elimination
	1,1-DCA	Hydrogenolysis
1,1-DCA	Ethane	Reductive α elimination
	Chloroethane	Hydrogenolysis

Reprinted from Arnold and Roberts (1999)

### Nanoscale iron

The study of Song and Carraway (2008) show that the situation is the reverse of that described by Janda et al. (2004), when nanoscale iron is present in water. The reaction rates of highly chlorinated ethenes are lower than those of low chlorinated ones, and no intermediate compounds are found in the water. The authors explain this by the catalytic activity of nanoscale iron, the reaction not being under reduction potential control. TCE degradation by nanoscale iron leads to formation of hydrocarbons (Liu et al. 2005). Another study by Song and Carraway (2005) investigated the conversion of chlorinated ethanes by nanoscale iron. Highly chlorinated ethanes are degraded faster in the presence of nanoscale iron. 1,1,1,2-Tetrachloroethane (1,1,1,2-TeCA) undergoes largely β elimination with 1,1-DCE as the main product; dehydrogenation to TCE accounted for only 5% of the total 1,1,1,2-TeCA disappearance. 1,1,2,2-TeCA also undergoes mainly β elimination leading to c-DCE (73%) and t-DCE (27%). The greater removal of 1,1,2,2-TeCA, compared to 1,1,1,2-TeCA, takes place via hydrodehalogenation, mainly due to the slower β elimination rates. 1,1,1-TCA was rapidly hydrogenolysed to form 1,1-DCA, whereas 1,1,2-TCA was removed much more slowly with ethane as the sole product. 1,1-DCA was slowly degraded by α elimination to form ethane, and no transformation of 1,2-DCA in the presence of nanoscale iron over period of 40 days was observed. Magnetite inhibits activity of nanoscale Fe^0^ towards 1,1,1-TCA dechlorination. Nanoscale iron reduced Fe^III^ compounds on magnetite surface instead of reductively dechlorinate 1,1,1-TCA (Bae and Lee 2010). However, nanoscale Fe^0^ exhibits inhibitory properties towards dechlorinating bacterial communities at concentrations of 0.3 g L^-1^, but at concentrations of 0.01–0.1 g L^−1^ bacterial-mediated dechlorination rates were lower (Barnes et al. 2010). Iron nanoparticles react with bacterial membranes, physically coat the cells and cause oxidative stress (Diao and Yao 2009). Coating nanoscale iron with olefin maleic acid copolymer, apart from increasing iron mobility in water, decreases the inhibitory effect towards dechlorinating bacteria (Xiu et al. 2010). Nanoparticles coated with carboxymethyl cellulose enhanced microbial acivity, hydrogen from iron corrosion served as electron donor, while carboxymethyl cellulose served as caron source (He et al. 2010a). Iron nanoparticles degrade TCE on the NAPL interphase and within NAPL itself, but the reactions are slower than those within aqueous phase. TCE degradation rates are proportional to water content in NAPL (Berge and Ramsburg 2010). In situ dechlorination of TCE took a few days, while nanoparticles remained active for 4–8 weeks (Zhang 2003). Application of bicomponent iron/silicon system promotes reactivity, results in low yield of chlorinated products and high hydrogen production (for bacterial growth stimulation; Doong et al. 2003). Also application of bimetallic iron/nickel nanoparticles immobilised in membrane has been reported. 20% of nickel content in the nanoparticles occurred to be the most effective in TCE dechlorination (Parshetti and Doong 2009).

### Palladium nanoparticles

Bimetallic nanoparticles—palladium deposited on iron are more efficient in dechlorination of ethenes than conventional iron or iron nanoparticles (Lien and Zhang 2007). This is due to high surface activity increased by catalytic activity of palladium (Lien and Zhang 2001). Chlorinated ethanes and ethenes are degraded rapidly, PCE and TCE are dechlorinated faster than 1,1,1-TCA. 1,1,1-TCA is degraded more slowly probably due to location of three chlorine atoms on one carbon atom (Cho and Choi 2010). The 1–5% palladium content in nanoparticles (concentration 5 g L^−1^) is the most efficient in dechlorination of TCE (Lien and Zhang 2007). Tin is considered to be effective in degradation of chlorinated ethanes and ethenes, therefore tin/palladium nanoparticles are also used. Tin appears to be more stable than iron in groundwater conditions (Lin et al. 2009).

Palladium encapsulated in alginate, polyurethane or polyacrylamide nanoparticles in batch reactor are able to dechlorinate 100 mg L^−1^ TCE in one hour. The main product is ethane with low concentrations of VC, 1,1-DCE, 1,2-DCE and 1,1-DCA. These by-products were completely dechlorinated after 24 h (Hennebel et al. 2009). In situ dechlorination by iron–palladium nanoparticles is also rapid (Bennett et al. 2010). Coating iron–palladium nanoparticles with carboxymethyl cellulose prevents formation of particle aggregates, thus increases mobility of nanoparticles in groundwater (He et al. 2010a).

Palladium-on-gold nanoparticles are catalytically more active than pure Pd itself. The reaction rates were higher for ethenes containing less chlorine atoms (Wong et al. 2009). No build up of daughter products has been observed. The presence of gold in nanoparticles slows the process of palladium deactivation by chlorides and sulphates (Wong et al. 2009).

Nanoparticles can be delivered to the contaminated groundwater by direct injection by gravity flow (Lien and Zhang 2007). However, they can be a potential environmental risk (Bystrzejewska-Piotrowska et al. 2009). The complete environmental risk assessment of releasing nanoparticles into environmental water is not available yet. Little is known about their ecotoxicity, bioaccumulation potential and persistency in the environment (Grieger et al. 2010).

### Green rusts

Green rusts are corrosion products of iron or steel and may occur naturally in reductomorphic soils. They are mixed Fe^II^ and Fe^III^ hydroxides, with chemical structure [Fe_6-x_^II^Fe_x_^III^(OH)_12_]^x+^[(*A*)_x/n_yH_2_O]^x−^, where *A* is an anion, typically SO_4_^2−^or Cl^−^. The degradation of PCE, TCE, c-DCE and VC is slow and results in the formation of acetylene and ethylene (Lee and Batchelor 2002a). Highly chlorinated ethanes are reduced in the presence of green rusts more rapidly than less substituted ones. Compounds with a more asymmetrical structure are also degraded more rapidly (i.e. 1,1,1,2-TeCA > 1,1,2,2-TeCA or 1,1,1-TCA > 1,1,2-TCA). The degradation of 1,2-DCA was not observed in the presence of green rusts (O’Loughlin and Burris 2004). The formation of green rusts may explain the long-term effectiveness of iron-based remediation techniques, although films of oxides are formed on the surface of iron.

### Sulphide minerals

Minerals like pyrite, magnetite and green rusts are able to degrade chlorinated ethanes and ethenes. Liang et al. (2009) stated that chloride green rust and pyrite are more effective in PCE and TCE degradation than sulphate green rust, magnetite and goethite. Mackinawite (FeS) has been found effective in reducing PCE and TCE under anoxic conditions with acetylene as the main product. The other products of TCE transformation are 1,1-DCE, c-DCE, ethene and ethane. TCE is a product of PCE hydrogenolysis in the presence of FeS. The presence of metals can influence the rates of PCE and TCE transformations: Fe^II^ or Ni^II^ reduce PCE and TCE transformation rates but Hg^II^ and Co^II^ do not (Jeong et al. 2007). This has been explained by the susceptibility of the β elimination transformation pathway to metal amendments. The differences in reaction rates are explained by the formation of the second sulphide phases (sulphide of given metal amendment) of different properties. Cobalt increases the transformation rates due to formation of Fe-coprecipitated CoS, while Ni is expected to form sulphides structurally closely associated to FeS, giving the effect of decreased transformation rates (Jeong and Hayes 2007).

Other studies (Butler and Hayes 1999) show that the main products of PCE and TCE transformation in the presence of FeS are acetylene and c-DCE. He et al. (2010b), in addition to these products, obtained ethane and methane (expected to be formed from radical intermediates) as minor products. PCE and TCE are converted by β elimination and hydrogenolysis, but β elimination is much faster (Jeong et al. 2007). The rate of TCE transformation increases with increasing pH (He et al. 2010b). 1,1,1-TCA was degraded to ethylene and 1,1-DCA, which suggests that hydrogenolysis is the main transformation pathway (Choi et al. 2009). Other studies show that 1,1-DCA and 2-butyne (from coupling of radical intermediates) are 1,1,1-TCA degradation products (Gander et al. 2002; Fennelly and Roberts 1998). The rates of reactions were increased in the presence of Co and Ni as amendments, probably because of their catalytic effects. The presence of sulphides, which is known to increase the reactivity of metals, resulted in an increase of degradation rates (Choi et al. 2009).

Mackinawite may slowly oxidise to pyrite in the presence of S^0^ and to iron oxides under more oxidising conditions (He et al. 2010b). Although pyrite is a ubiquitous mineral in anaerobic environments, it degrades chlorinated ethanes and ethenes more slowly than mackinawite. Surface area-normalised pseudo-first-order initial rate constants (Lm^−2^ day^−1^) for PCE reductive dechlorination are 1.97 × 10^−5^ by pyrite and 2.74 × 10^−2^ by mackinawite (Lee and Batchelor 2002b). Pyrite degrades chlorinated ethanes and ethenes via β elimination and hydrogenolysis. Lee and Batchelor (2002b) state that chlorinated compounds are adsorbed at reactive sites, where dechlorination occurs by pseudo-first-order kinetics, depleting reactive sites and hence the reductive capacity of the mineral. The results of Weerasooriya and Dharmasena (2001) show that the reductive dechlorination of TCE in the presence of pyrite (like FeS) occurs faster in an alkaline environment. The main products of the pyrite-assisted dechlorination of PCE, TCE, c-DCE and VC are acetylene and ethylene (Lee and Batchelor 2002b). Under aerobic conditions, in the presence of pyrite (FeS_2_), the conversion pathway of TCE is different from reductive dechlorination. Pyrite undergoes a Fenton-like reaction in the presence of oxygen, forming a hydroxyl radical, a strong oxidant capable of oxidising many organic compounds. The products of TCE oxidation in the presence of pyrite and oxygen are carbon dioxide and chloride; the reaction rates increased from 0.004 to 0.013 h^−1^ with the increase of concentration of oxygen in water from 0.017 to 0.268 mmol (Pham et al. 2008). Organic compounds can be adsorbed on the surface of mineral and affect the degradation of chlorinated compounds (He et al. 2009). Lee and Batchelor (2002b) extrapolated iron sulphides laboratory results of reductive dechlorination to environmental conditions: they calculated that the degradation half-life of PCE in the presence of pyrite and magnetite is 13 and 608 days, respectively. Magnetite may be capable of reduction of DCEs to acetylene without VC production (Ferrey et al. 2004).

Biogeochemical reductive dechlorination (BiRD) is interesting method of chlorinated ethanes and ethenes remediation. Sulphate-reducing bacteria produce hydrogen sulphide in reaction of organic matter with sulphates. Hydrogen sulphide react with iron oxides present in the sediments, to form iron sulphides, which are known to dechlorinate chlorinated ethanes and ethenes (Kennedy et al. 2006a). BiRD is stimulated by addition sulphate and organic carbon. Iron, typically mineral, is usually naturally present in the aquifer but might be also supplemented (Kennedy et al. 2006b). The advantages of BiRD are rapid dechlorination and no generation of less-chlorinated daughter products (Kennedy et al. 2006a).

Other sulphides are capable of reducing PCE and TCE. CoS is very effective in removing PCE and TCE (both were degraded so fast it was impossible to determine rate constants) via reductive dechlorination. In the presence of NiS (rates are (6.09 ± 0.77) × 10^−4^ for PCE and (8.84 ± 0.59) × 10^−4^ for TCE) such a reaction also takes place but it is slower than for FeS (rates are (7.60 ± 1.01) × 10^−4^ for PCE and (2.12 ± 0.1) × 10^−3^ for TCE); HgS showed no ability to convert PCE or TCE (Table 3; Jeong and Hayes 2007).

**Table 3:** Reductive dechlorination degradation products and rate constants mediated by different metals

Metal or metal compound + amendments	Surface area concentration (m^2^ L^−1^)	Substrate	Products	Surface area normalised pseudo-first-order rate constant (L m^−2^ h^−1^)	Reference
Fe^0^	340	PCE	TCE	7.4 × 10^−6^	Dayan et al. (1999)
		TCE	Dichloroethenes	2.5 × 10^−5^	
		c-DCE	VC	1.6 × 10^−5^	
	not specified (10 g L^−1^)	PCE	Less chlorinated	3.2 × 10^−3a^	Ma and Wu (2008)
	39	TCE	Acetylene, dichloroethenes	1.2 × 10^−4^	Hara et al. (2005)
Fe^0^	2.8	PCE	Ethane, ethylene	3.4 × 10^−3^	Doong and Lai (2006)
Fe^0^ + humic acid				5.4 × 10^−4^	
Fe^0^ + Cu^II^				8.2 × 10^−3^	
Fe^0^ + Cu^II^ + humic acid				1.7 × 10^−3^	
Fe^0^ + Co^II^				6.1 × 10^−3^	
Fe^0^ + Co^II^ + humic acid				2.2 × 10^−3^	
Fe^0^ + Ni^II^				2.9 × 10^−1^	
Fe^0^ + Ni^II^ + humic acid				9.3 × 10^−2^	
Nano-Fe^0^	2.2	1,1,1,2-TeCA	Ethane, ethylene, 1,1-DCE	5.5 × 10^−1^	Song and Carraway (2005)
		1,1,2,2-TeCA	Ethane, c-DCE	3.1 × 10^−2^	
		1,1,1-TCA	Ethane, 1,1-DCA	1.5 × 10^−1^	
		1,1,2-TCA	Ethane	2.4 × 10^−3^	
		1,1-DCA	Ethane	1.1 × 10^−4^	
Nano-Pd/Fe	0.44	PCE	Ethene, ethane	1.2 × 10^−2^	Lien and Zhang (2001)
		TCE		1.8 × 10^−2^	
		t-DCE		1.5 × 10^−2^	
		c-DCE		1.8 × 10^−2^	
		1,1-DCE		1.2 × 10^−2^	
FeS_2_	578	PCE	Ethylene, c-DCE, TCE	1.6 × 10^−6^	(Liang et al. 2009)
		TCE	Ethylene, c-DCE	6.4 × 10^−5^	
	2	TCE	Acetylene	8 × 10^−3^	Weerasooriya and Dharmasena (2001)
FeS_2_ + oxygen	20	TCE	CO_2_, Cl^-^	5 × 10^−4^	(Pham et al. 2008)
FeS	not specified (33 g L^-1^)	1,1,1-TCA	ethylene, 1,1-DCA	3.7 × 10^−2a^	(Choi et al. 2009)
	not specified (10 g L^-1^)	PCE	Ethylene (minor amounts of 1,1-DCE and c-DCE)	9.4 × 10^−4a^	Jeong et al. (2007)
		TCE		2.2 × 10^−3a^	
	0.5	PCE	Acetylene, c-DCE	8 × 10^−4^	Butler and Hayes (1999)
		TCE		2.4 × 10^−3^	
FeS + cysteine		PCE		2.2 × 10^−4^	
		TCE		7 × 10^−4^	
FeS + Fe^II^	not specified (10 g L^-1^)	PCE	Ethylene (minor amounts of 1,1-DCE and c-DCE)	6.7 × 10^−4a^	Jeong et al. (2007)
		TCE		9.9 × 10^−4a^	
FeS + Co^II^		PCE		8.6 × 10^−3^	
		TCE		4.3 × 10^−3^	
FeS + Ni^II^		PCE		6.3 × 10^−4^	
		TCE		3.1 × 10^−4^	
FeS + Hg^II^		PCE		1.7 × 10^−3^	
		TCE		1.3 × 10^−3^	
Fe (hydr)oxide	not specified	PCE	Acetylene,c-DCE, 1,1-DCE	2.5 × 10^−3a^	Jeong and Hayes (2007)
		TCE		9.2 × 10^−3a^	
NiS	not specified	PCE	Acetylene	6.1 × 10^−4a^	
		TCE		8.8 × 10^−4a^	
Chloride green rust	210	PCE	Acetylene, ethylene, TCE	5.6 × 10^−6^	Liang et al. (2009)
		TCE	Acetylene, Ethylene, c-DCE	2.9 × 10^−5^	
Sulphate green rust	604	PCE	Acetylene, ethylene	6.7 × 10^−6^	Lee and Batchelor (2002a)
		TCE		3.6 × 10^−6^	
		c-DCE		2.2 × 10^−6^	
		VC		3.2 × 10^−6^	
Zn	1.4	PCE	TCE, t-DCE, acetylene	3 × 10^−1^	Arnold and Roberts (1998)
	7	TCE	t-DCE, acetylene, c-DCE	2.2 × 10^−3^	
		t-DCE	Acetylene, ethylene, VC	1.4 × 10^−5^	
		c-DCE	Acetylene, VC	3.5 × 10^−6^	
		1,1-DCE	VC	4.1 × 10^−6^	
		VC	Ethylene	10^−4^	
	0.7	Dichloroacetylene	Chloroacetylene, t-DCE	25	
	0.14	Chloroacetylene	Acetylene, VC	7	
	Not specified (10 g L^-1^)	PCE	Less chlorinated	9.6 × 10^−3a^	Ma and Wu (2008)
Zn + hydroxyapatite	1.37	PCE	TCE, t-DCE	2 × 10^−1^	Song et al. (2008)

^a^Observed rate constants (per hour)

### Porphyrins

Another group of compounds capable of reducing chlorinated ethanes and ethenes are porphyrins. Found in subsurface waters, they are excellent reductive catalysts under a variety of conditions. They are produced by the variety of microorganisms in anaerobic-light, aerobic and iron-deficient conditions (Utsunomiya et al. 2003). The abiotic vitamin-mediated degradation (heat-killed microorganisms) is four to five times lower than corresponding microbiological degradation (Guerrero-Barajas and Field 2005). The studies of Dror and Schlautman (2004a) show that nickel, iron and vanadium oxide porphyrins (commonly found in water) are non-reactive in pure water, but they catalyse dechlorination in the presence of co-solvents, such as dimethyl formamide. Increasing porphyrin solubility is key factor for porphyrin mediated reductive dechlorination of chlorinated solvents. Vitamin B_12_ (cobalt containing natural porphyrin) is a catalyst of dechlorination reactions in water without co-solvent, with titanium citrate as reductant. The mechanism of reductive dechlorination of chlorinated solvents involves formation of bond between the metal and chlorinated solvent carbon atom. The reduced porphyrin transfers the electron to chlorinated solvent molecule, then oxidised porphyrin is reduced by the reductant present in the environment. More detailed model involves the role of macrocycle, not only core metal, in the reaction. The macrocycle possesses conjugated π system that allows electron transfer also through the macrocycle itself, not only by the metal (Dror and Schlautman 2003). The main abiotic dechlorination products of PCE catalysed by vitamin B_12_ are acetylene and ethylene, which are formed through TCE and c-DCE (McCauley et al. 2005). The reduction of PCE occurs via hydrogenolysis and β elimination as competing pathways (Burris et al. 1996), with the build-up of c-DCE as daughter compound (Burris et al. 1998).

**Fig. 2:**
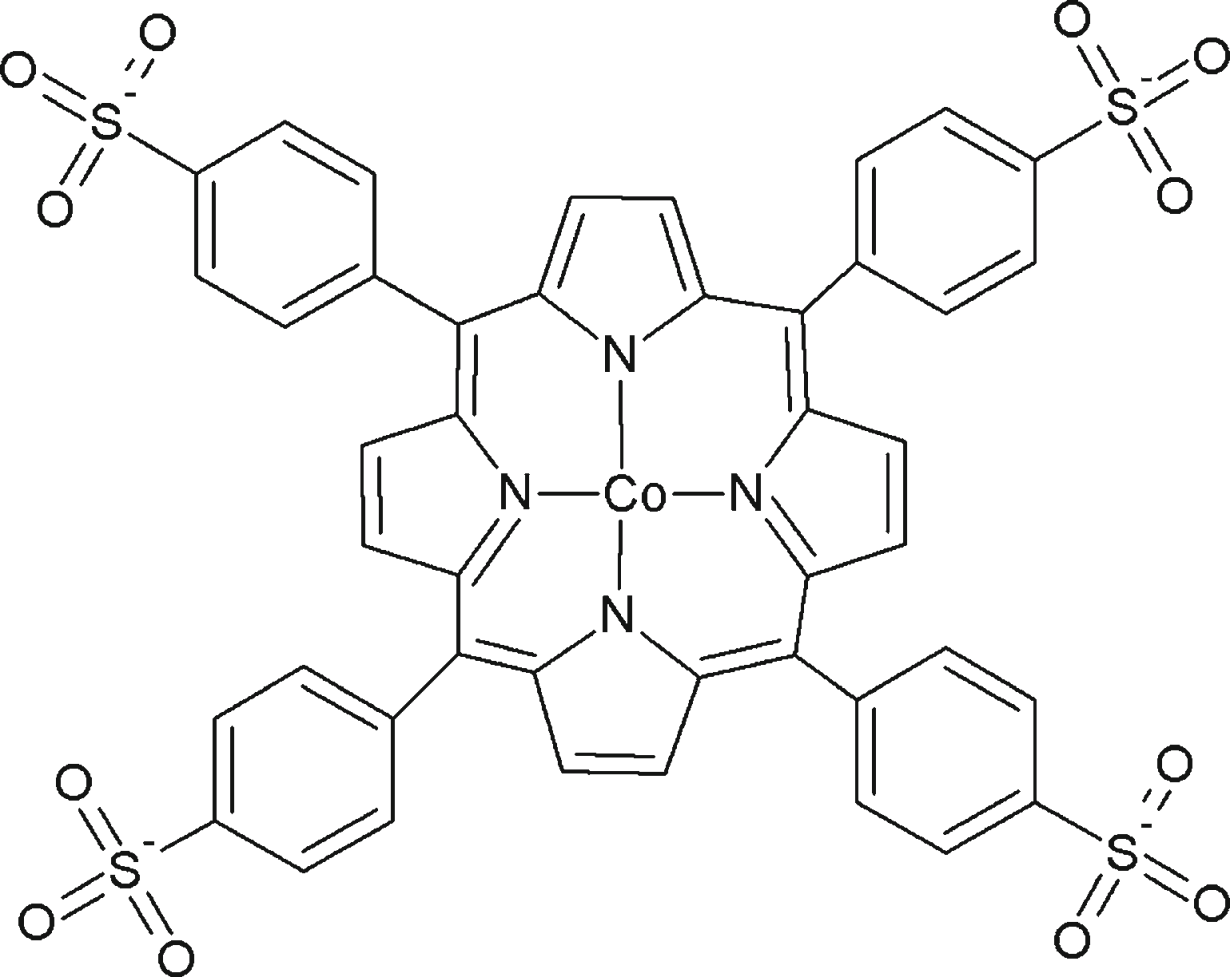
Chemical structure of tetrakis-(4-sulphonatophenyl)porphyrin cobalt

Porphyrins are similar in structure to vitamin B_12_ 5,10,15,20-tetrakis(4-carboxyphenyl)porphyrin cobalt ((TCPP)Co) in the presence of a reducing agent catalyses PCE and TCE degradation through c-DCE to hydrocarbons. Degradation rates were higher at elevated pH and at higher concentrations of reducing agent (Fritsch and McNeill 2005). The degradation rates of TCE in the presence of tetrakis-(4-sulphonatophenyl)porphyrin cobalt (TSPP)Co (see Fig. 2) are three times higher compared to (TCPP)Co, whereas dechlorination rates for PCE are similar (Barnett et al. 2010). Porphyrin-catalysed dechlorination reaction rates may be very high (up to 10^−2^ h^−1^) even for micromolar catalyst concentrations. The most effective catalysts of reductive dechlorination seem to be Co and Ni containing tetrakis (*N*-methyl-4-4-pyridiniumyl) porphyrin (Dror and Schlautman 2004b). Porphyrins catalyse reductive dechlorination of chlorinated solvents when present in solution as well as when immobilised on solid surfaces (Burris et al. 1996).

Also, other naturally occurring compounds may stimulate reductive dechlorination of chlorinated solvents. Quinones can increase reduction rates by iron, due to the effect of electron transfer mediation (Tratnyek et al. 2001). Similarly, quinones catalyse reduction by hydrogen sulphide (Uchimiya and Stone 2009). Humic substances can form Ni and Cu complexes to that effectively transfer electrons in reductive dechlorination reactions in the presence of bulk reductant (O’Loughlin et al. 1999).

## Oxidation

Chlorinated ethanes and ethenes cane be converted to nontoxic compounds by oxidation. Oxidising agents used in remediation of chlorinated ethanes and ethenes are potassium permanganate, Fenton’s reagent, ozone, chlorine dioxide (Kao et al. 2008).

### Permanganate

Potassium permanganate (KMnO_4_) is strong oxidant that can be successfully applied in remediation of chlorinated ethanes and ethenes. TCE redox reaction with permanganate may be presented as (Kim and Gurol 2005):

5 $$ {\text{C}}{{\text{l}}_{{2}}}{\text{C}} = {\text{CHCl}} + {\text{2Mn}}{{\text{O}}_{{{{4}^{ - }}}}} \to {\text{2 C}}{{\text{O}}_{{2}}} \uparrow + {\text{2 Mn}}{{\text{O}}_{{2}}} \downarrow + {\text{3C}}{{\text{l}}^{ - }} + {{\text{H}}^{ + }} $$

Unlike with reductive dechlorination, formation of toxic daughter compounds is avoided. Higher removal efficiency is observed under acidic conditions than alkaline (Kao et al. 2008). The degradation rates are inversely propotional to the number of chlorine atoms in ethene molecule. *Cis*-isomers are less stable than *trans*-isomers (Yan and Schwarz 1999). Chlorinated ethanes and ethenes in dissolved phase are oxidised rapidly, however removal of DNAPL chlorinated solvent is slow process limited by mass transfer towards water–DNAPL interphase (Huang et al. 2002). Oxidation of chlorinated ethanes and ethenes with MnO_4_^−^ can be enhanced by transferring MnO_4_^−^ to the DNAPL by phase-transfer catalysis. This can be obtained by the application of pentyltriphenylphosphonium cation instead of potassium (Seol and Schwartz 2000). Oxidation of DNAPL with MnO_4_^−^ may be enhanced by application of co-solvent that increases water solubility of DNAPL. Therefore, acetone and *tert*-butyl alcohol are applied together with KMnO_4_^−^ (Zhai et al. 2006). Another approach to increase solubility of DNAPL is application of surfactants. Chlorinated ethanes and ethenes are trapped in micelles which are more dispersed in water than DNAPL leading to MnO_4_^−^ mass transfer improvement (Tsai et al. 2009).

Formation of CO_2_ gas and MnO_2_ precipitate (see reaction 5) is undesirable process in porous media as this process may deteriorate hydraulic properties of the soil and prevent delivery of MnO_4_^−^ to DNAPL (Schroth et al. 2001).

### Fenton process

Fenton’s reagent is a mixture of ferrous salts and hydrogen peroxide. In reaction 6, they react to form hydroxyl radical, hydroxyl anion and in further reactions hydroperoxyl radical (Chen et al. 2001).

6 $$ {\text{F}}{{\text{e}}^{{{2} + }}} + {{\text{H}}_{{2}}}{{\text{O}}_{{2}}} \to {\text{F}}{{\text{e}}^{{{3} + }}} + {\text{O}}{{\text{H}}^{ - }} + {\text{O}}{{\text{H}}^{ \bullet }} $$

**Fig. 3:**
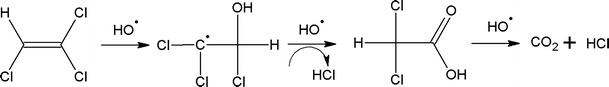
TCE oxidation with hydroxyl radical

Hydroxyl and hydroperoxyl radicals are able to oxidise broad spectrum of organic pollutants, including chlorinated ethanes and ethenes. TCE is oxidised with hydroxyl radical through dichloroacetic acid. Figure 3 presents TCE oxidation pathway (Qiang et al. 2008). Chlorinated ethenes are easily oxidised by hydroxyl radical due to the presence of double bond in their structure.

The main limitation of Fenton’s reagent in situ application is fast precipitation of iron in form of iron hydroxide. To prevent precipitation iron complexes are used, as well as zero-valent iron or iron sulphide are used as iron source (Che and Lee 2011). The pyrite Fenton reaction produced simultaneously hydroxyl radical (oxidant) and superoxide anion radical (reductant). Hexachloroethane (saturated, perchlorinated compound) is degraded *via* reaction with reductant, while PCE is degraded mainly via oxidative pathway (Jho et al. 2010). At some sites, no addition of iron would be required as naturally occurring iron-bearing minerals can react with hydrogen peroxide to produce hydroxyl radical (Teel et al. 2001).

## Summary

There are many chemical agents naturally occurring in water that can mediate the dechlorination reactions of chlorinated ethanes and ethenes. Their mutual relations are very complex, and because these agents are usually present at low concentrations, degradation reaction rates are slow. Chlorinated ethanes and ethenes present in environmental water in favourable conditions may be degraded to harmless products. Under less favourable conditions, however, dechlorination does not proceed at all or may be incomplete, leading to the formation of more toxic daughter compounds. In such cases, natural attenuation processes can be enhanced by in situ remediation with the addition of zero-valent metals, sulphide minerals or oxidation agents. Choosing the proper remediation strategy has to be preceded by an exhaustive site examination in order to fit the best strategy to the given site conditions.

There are still gaps in our understanding of the degradation of chlorinated ethanes and ethenes in water. Future investigations should therefore focus on the dechlorination of compounds in complex, multicomponent systems and mutual relations between microbial and abiotic degradation. Iron or bimetallic nanoparticles are very effective in removal of chlorinated ethanes and ethenes, however more research is needed to understand risk of their release to the aquifer. Since abiotic degradation agents can successfully degrade chlorinated ethanes and ethenes at high concentrations or in DNAPL itself, future research should also focus on efficient transfer of dechlorination agent to DNAPL or DNAPL–water interphase.
